# Modulation of primary human apical papilla stem cells: Influence of *Enterococcus faecalis*, oxygen levels, and calcium silicate‐based cements

**DOI:** 10.1111/eos.70025

**Published:** 2025-06-13

**Authors:** Olena Rakhimova, Valeriia Zymovets, Lahood Abdalla, Bagir Soltani, Malin Brundin, Peyman Kelk, Nelly Romani Vestman

**Affiliations:** ^1^ Department of Odontology Umeå University Umeå Sweden; ^2^ Department of Medical and Translational Biology Umeå University Umeå Sweden; ^3^ Wallenberg Centre for Molecular Medicine Umeå University Umeå Sweden

**Keywords:** alkaline phosphatase, biodentine, endodontics, mineral trioxide aggregate; MTA

## Abstract

Stem cells from the apical papilla (SCAP) are essential for regenerative endodontic treatment. Although mineral trioxide aggregate (MTA) and Biodentine are widely used in regenerative endodontic treatment procedures, their effects on SCAP remain unclear. This study investigated the impact of ProRoot MTA and Biodentine on SCAP viability and mineralization in the presence of *Enterococcus faecalis* under aerobic and anaerobic environments. Stem cells from the apical papilla were isolated from three healthy donors and exposed to three different surface area‐to‐volume (SA:V) ratio extracts of ProRoot MTA and Biodentine for 21 days in aerobic or anaerobic conditions. Cell viability was assessed using a neutral red cytotoxicity assay, and mineralization was evaluated by measuring alkaline phosphatase (ALP) activity. No significant differences between ProRoot MTA and Biodentine regarding SCAP viability were detected; however, increased cytotoxicity was found (for both ProRoot MTA and Biodentine) at the highest SA:V ratio of extract used. Oxygen availability, as well as variability in responses of SCAP from the different donors, resulted in greater variation of ALP levels than did type of material. Both ProRoot MTA and Biodentine showed comparable effects on SCAP viability and mineralization, with high SA:V ratios of extracts resulting in increased cytotoxicity. Mineralization in SCAP is influenced by oxygen conditions and the presence of *E. faecalis*, elucidating the need for further in vivo studies to optimize regenerative endodontic treatment outcomes.

## INTRODUCTION

Traumatic dental injuries are prevalent and rank fifth among the world's most frequent diseases and injuries, affecting approximately one in seven people globally [1]. Pulp necrosis, which occurs in 26.9% of all traumatic dental injury cases [2], is the most common complication of traumatic dental injuries, leading to short roots, thin dentin walls, and an increased risk of cervical fracture in permanent teeth [3]. Endodontic treatment of such patients is challenging and may prolong treatment time [4].

Currently, apexification techniques using calcium hydroxide or mineral trioxide aggregate (MTA) are used to stimulate the formation of a calcified barrier that encloses an open apical area [5]. Although calcium hydroxide aids in the preparation of immature necrotic teeth for endodontic treatment, prolonged exposure increases the risk of root fracture [6], and no technique has been shown to improve long‐term root formation [7]. An alternative technique is regenerative endodontic treatment, which aims to regenerate the dentin–pulp complex with further root development and a potentially vital pulp response [8]. In regenerative endodontic treatment, calcium‐enriched materials, such as MTA or Biodentine, are utilized as barriers [9]. Mineral trioxide aggregate is a hydraulic calcium silicate cement primarily composed of Portland cement, tricalcium silicate, dicalcium silicate, and bismuth oxide. It hardens upon contact with water [10] and seals the root canal system effectively [11]. The characteristics of MTA that are essential for regenerative endodontic treatment include having a high pH (as this is antibacterial and antifungal), being radiopaque, and having the capacity to form a strong bond with dental materials [12].

Biodentine is a bioactive calcium silicate‐based cement in which zirconium oxide is commonly used as a radiopacifier [13] in treatments such as vital pulp therapy, repair, apexification, and regenerative endodontic treatment because of its similarity to MTA [14]. Compared with MTA, Biodentine has a shorter setting time and greater strength [15].

In addition to the importance of the materials used in regenerative endodontic treatment for barrier creation, stem cells, growth factors, and extracellular matrix scaffolds are crucial for successful tissue regeneration [16]. In this context, the apical papilla of immature human permanent teeth harbors a specific type of mesenchymal stem cell (MSC): stem cells from the apical papilla (SCAP) [17]. Stem cells from the apical papilla are particularly valuable because of their high proliferative capacity and their ability to form odontoblast‐like cells and to produce dentin [18], making them highly favorable in the regenerative endodontic treatment field. However, the presence of bacteria or bacterial infections can hinder the success of regenerative endodontic treatment, even when other critical components are present [19, 20, 21, 22]. We have previously demonstrated that bacterial modulation of SCAP affects their viability, immune responses, and the expression of genes related to osteogenesis and dentinogenesis [23, 24]. Moreover, *Enterococcus faecalis* has been associated with failure of root canal treatment [25, 26, 27, 28] and can alter the expression of genes involved in dentin and bone formation [23]. Several studies have revealed that oxygen levels may modulate stem cell responses [29, 30, 31, 32, 33, 34]; however, the extent to which oxygen levels modulate the responses of SCAP in the presence of bioactive materials remains unclear.

Considering the widespread clinical use of Biodentine and ProRoot MTA, further research is essential to understand their critical and inevitable effects on SCAP under various conditions. This study aimed to investigate the cytotoxicity and mineralization of SCAP in response to ProRoot MTA and Biodentine, particularly in the presence of *E. faecalis*, under aerobic and anaerobic conditions.

## MATERIAL AND METHODS

### Preparation and treatment of calcium silicate‐based cements

In this study, ProRoot MTA White (Dentsply Tulsa Dental) and Biodentine (Septodont) were used. The cements were mixed aseptically according to the manufacturers’ instructions, shaped into discs 5 mm in diameter and 3 mm in height, and sterilized under ultraviolet irradiation (UV) (100–280 nm) for 30 min.

To ensure consistency and reliability of results, the size and shape of the test samples were standardized. ProRoot MTA and Biodentine were prepared in discs (as mentioned above) according to ISO 10,993‐12, with a surface area‐to‐volume (SA:V) ratio of 3 cm^2^/mL [35]. To evaluate different concentrations of ProRoot MTA and Biodentine, these cements were prepared in discs at three SA:V ratios: 1.5, 3, and 6 cm^2^/mL. Then, the discs were placed in 15 mL of sterile Minimum Essential Medium alpha modification (MEM‐α) with GlutaMAX (Thermo Fisher Scientific, #32561‐094), supplemented with 10% fetal bovine serum (FBS) (Sigma Aldrich, #F7524) and 1% Antibiotic‐Antimycotic Solution (Sigma Aldrich, #P0781), and incubated for 24 h at 37°C on a rotating platform. Sarstedt 15 mL tubes (Sarstedt; Cat. No. 62.554.002) were used for incubation of the discs. The solutions containing extracts from ProRoot MTA and Biodentine discs were then double‐filtered through a 0.22 µm filter (VWR International, #103573‐246) to remove any remaining particles.

### Stem cells and culture conditions

In this study, we used SCAP (limited to passages 5 and 8) previously isolated from three healthy donors (Donors I, II, and III) [36, 37]; the study was approved by the Ethics Committee of Umeå University (Reg. no. 2013‐276‐31 M). The SCAP for these studies were taken from the cryostocks and then cultured in MEM‐α (Sigma Aldrich) with 10% FBS (Sigma Aldrich) and 1% Antibiotic–Antimycotic Solution (Sigma Aldrich) at 37°C in a 5% CO_2_ and humidified atmosphere. Before the experiments, the SCAP were synchronized by starvation for 18 h in 1% FBS. The medium was refreshed every other day.

The SCAP from Donors I, II and III were seeded in 96‐well, flat‐based plates (Sarstedt; Cat. No. 83.3924) at 2.5 × 10⁴ cells per well in triplicate for each variant of treatment, including controls. After overnight adhesion in a CO₂ incubator at 5% CO_2_ and 37°C, the cells were examined the following day using a Nikon TMS inverted phase contrast microscope. The cell culture medium containing unattached cells was subsequently replaced with 100 µL of solution prepared from discs containing ProRoot MTA or Biodentine at a SA:V ratio of 1.5, 3, or 6 cm^2^/mL. Two 96‐well plates were prepared: one was incubated aerobically (5% CO₂) and the other was incubated anaerobically (5% CO₂, 10% H₂, and 85% N₂) at 37°C.

### Coculture of SCAP, *E. faecalis*, and cement extracts

To investigate whether inactivated *E. faecalis* can affect interactions between SCAP and cement, we used UVC‐inactivated bacteria. Treatment of *E. faecalis* with UVC results in DNA damage, making the bacteria nonviable as they cannot grow or reproduce. Suspensions of UVC‐inactivated *E. faecalis* strain Tand‐4F [38] were applied to SCAP at a multiplicity of infection (MOI) of 100 (i.e. at a ratio of 100 bacteria to 1 SCAP cell) and cocultured for 21 d with a solution prepared from ProRoot MTA or Biodentine discs of 6 cm^2^/mL SA:V ratio. The cell culture medium was changed every 3 days.

### Neutral red cytotoxicity assay

A neutral red assay was used to assess the viability of SCAP after exposure to cement extracts and inactivated bacteria [39]. After 21 days of incubation, SCAP from Donors I, II, and IIIlocated in the 96‐well plates were washed with 150 µL of phosphate‐buffered saline (PBS) per well and incubated with 100 µL of 1% neutral red solution (stock concentration: 40 µg/mL) per well for 2 h at appropriate culture conditions. After incubation, the plates were washed with 150 microliters of PBS, then the washing solution was removed by gentle tapping. To each well 150 microliters of solubilization solution (50% ethanol, 49% Milli‐Q water, and 1% acetic acid) was added and exposed for 20 min at 20°C. The absorbance of obtained neutral red extract was measured at 540 nm using a microplate reader. The data were expressed in relative units of absorbance for each treatment variant, with background subtraction from empty (no cells) wells. Untreated SCAP were used as controls.

### ALP activity assay

The ALP test was used to examine the mineralization of SCAP in the presence of ProRoot MTA, Biodentine, and bacterial stimuli. The previously mentioned approach of two cell‐culture plate replicates was used in this experiment. The SA:V ratio of cement extract 6 cm^2^/mL that was found to be effective in the neutral red assay was applied to SCAP in 96‐well plates, which were incubated under aerobic or anaerobic conditions at 37°C for 21 days. In addition, SCAP cocultured with inactivated *E. faecalis* and cement extracts were incubated under anaerobic conditions. The cell culture medium was changed every 3 days.

The presence of ALP was visualized in SCAP (preliminarily fixed in 4% formalin) using Naphthol AS‐MX phosphate disodium salt (Sigma Aldrich, 855‐25ML) and Fast Blue BB (Sigma Aldrich, F0500‐25G) following standard histochemical procedures. The samples were examined using a Nikon TMS inverted phase contrast microscope, and the images were analyzed using the Trainable Weka Segmentation Plugin in Fiji to calculate the percentage of the area occupied by ALP. Data are expressed as the percentage of ALP‐positive areas, with the values for each SCAP donor cultured with Biodentine versus ProRoot MTA analyzed using a two‐way ANOVA.

### Statistical analysis

All experiments were performed using SCAP from three different donors, as biological replicates. The experiments were performed in triplicate for the SCAP from each donor. The results are expressed as mean ±  SD. Statistical analyses were performed using GraphPad Prism 7.0 (GraphPad Software). A value of *p* < 0.05 was considered statistically significant.

## RESULTS

### Effects of oxygen and different ratios of Biodentine and ProRoot MTA extracts on SCAP viability

**FIGURE 1:**
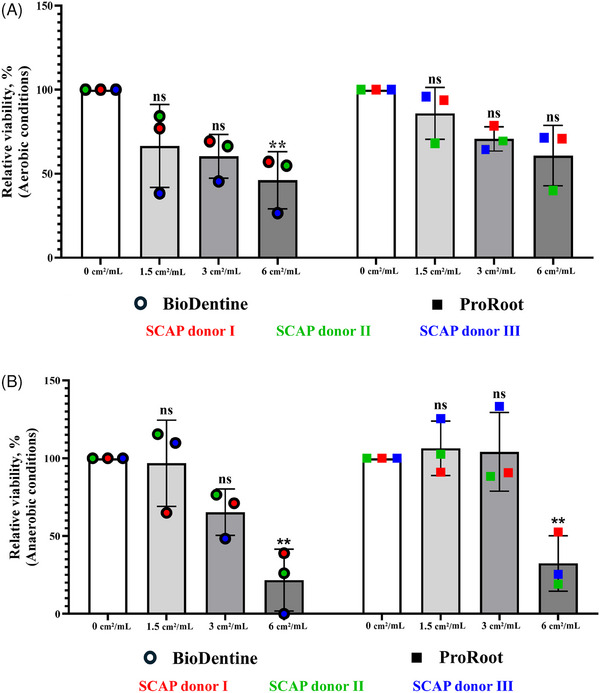
Viability of stem cells from the apical papilla (SCAP) cultured aerobically (A) and anaerobically (B) in different surface area‐to‐volume ratios (0, 1.5, 3, and 6 cm^2^/mL) of cement (ProRoot MTA and Biodentine) extracts. Each bar represents the mean of three separate biological samples (SCAP Donors I, II, and III) with three technical replicates at each variant of treatment, shown with SD. Data were analyzed using Tukey's multiple comparison test. Each viability value obtained for SCAP cultured with the designated cement extract ratio (1.5, 3, or 6 cm^2^/mL) was compared with the viability value obtained for SCAP cultured without extracts. **, statistically significant differences between values (*p* < 0.01). ns, nonsignificant results.

Viability of SCAP was influenced by both the type of cement and the environmental conditions (Figure 1). Under aerobic conditions (Figure 1A), exposure to increasing SA:V ratios of Biodentine extracts resulted in a dose‐dependent reduction in cell viability, with a 53.8% decrease observed at the highest SA:V ratio (6 cm^2^/mL, *p* < 0.01). By contrast, ProRoot MTA exhibited a more stable viability profile with no significant differences across the different ratios of extracts examined. Under anaerobic conditions (Figure 1B), the cytotoxic effects of Biodentine were more pronounced, with a 78.3% reduction in SCAP viability at a SA:V ratio of 6 cm^2^/mL (*p* < 0.01). Similarly, ProRoot MTA showed a 67.6% decrease at an SA:V ratio of 6 cm^2^/mL (*p* < 0.01), indicating that anaerobic conditions may enhance the cytotoxic effects of both materials. Notably, donor variability was observed across the experimental conditions, suggesting individual biological differences in SCAP responses to these materials.

As controls, SCAP were cultured without any treatment under both aerobic and anaerobic conditions to determine whether the absolute values could be used to normalize the data and compare the treatment effects on cell viability. The neutral red assay revealed no significant differences in SCAP viability between aerobic and anaerobic conditions (Figure S1).

The viability/cytotoxicity test data were analyzed using two‐way ANOVA. It was found that the SA:V ratio of cement affected SCAP viability under both aerobic (61.5%; *p* < 0.0003) and anaerobic (73.8%; *p* < 0.0003) conditions, regardless of oxygen availability.

To determine whether the viability of SCAP was affected differently by Biodentine and ProRoot MTA when used at similar SA:V ratios, data were analyzed using Sidak's multiple comparison test. No significant differences were found between the viability values (Table S1, S2).

### Effects of Biodentine and ProRoot MTA, presence of oxygen, and exposure to *E. faecalis* on production of ALP as a mineralization marker

**FIGURE 2:**
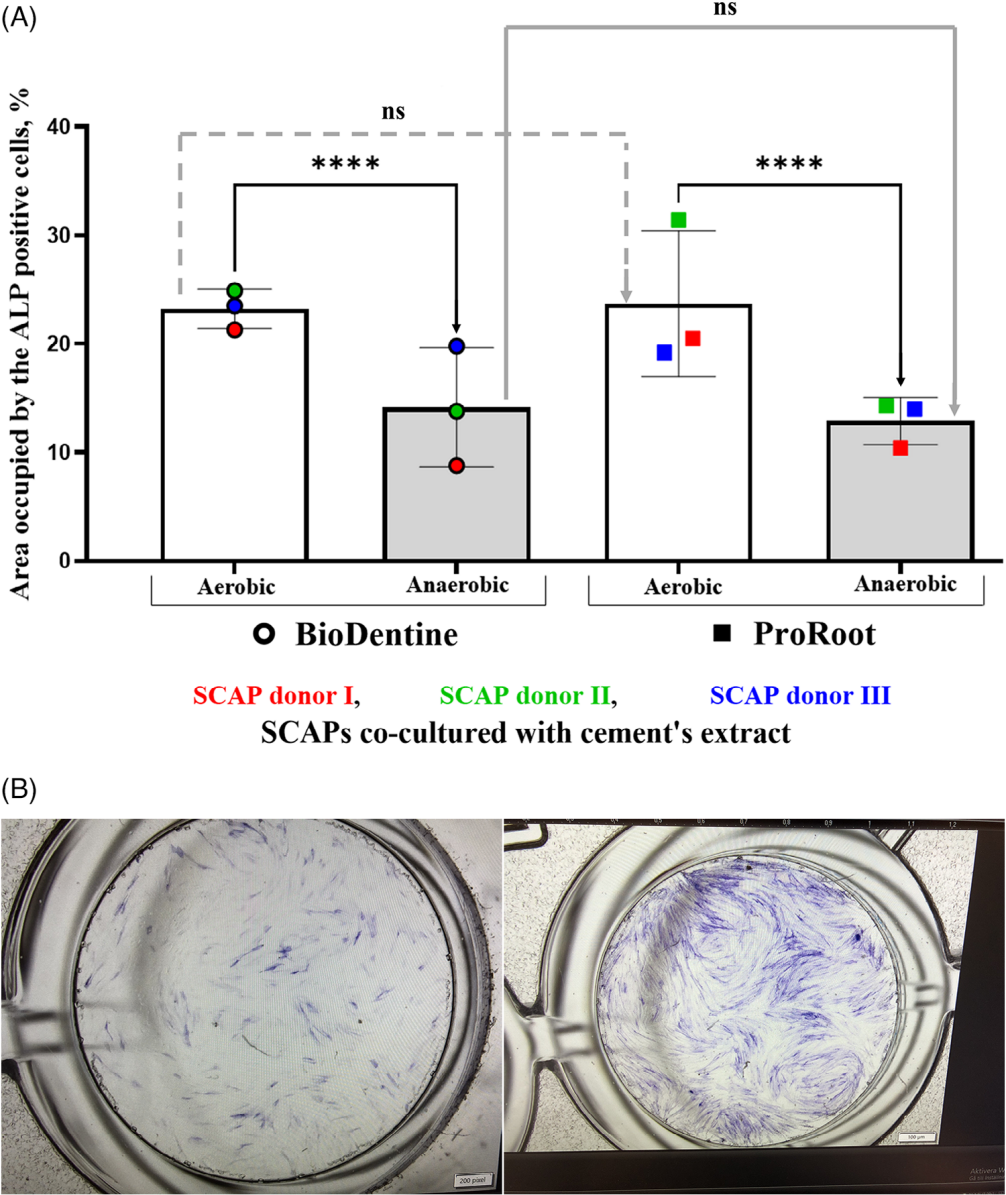
(A) Area occupied by alkaline phosphatase (ALP)‐positive stem cells from the apical papilla (SCAP) after culture with Biodentine or ProRoot MTA cement extracts (6 cm^2^/mL) under aerobic and anaerobic conditions. Each bar represents the mean of three biological replicates (SCAP Donors I, II, and III), with eight measurements taken per treatment condition, shown with SD. The ALP‐positive cell areas were compared using Tukey's multiple comparison test, revealing that oxygen availability caused statistically significant differences (**p < 0.0001). ns, nonsignificant results. (B) Representative images of SCAP histochemically stained for ALP in anaerobic versus aerobic culture conditions without application of cement material extracts.

As shown in Figure 2, the area occupied by ALP‐positive SCAP was notably influenced by oxygen availability. Under anaerobic conditions, ALP‐positive SCAP exposed to Biodentine and ProRoot MTA extracts showed a decrease in ALP expression of 9.1% and 10.8%, respectively, compared with cells exposed to these extracts under aerobic conditions (*p* < 0.0001). No significant difference was observed between Biodentine and ProRoot MTA under the same oxygen conditions (Figure S2A, B), suggesting that while both materials similarly support SCAP mineralization potential, oxygen availability plays a more critical role in regulating ALP activity.

Statistical analysis revealed significant variation in ALP levels between the SCAP donors, suggesting that the biological variation of the cell donors is the major contributor to mineralization under both aerobic (47.2%, *p* < 0.0001) and anaerobic (45.9%, *p* < 0.0001) conditions. Notably, analysis of the combined effects of cement type at an SA:V ratio of 6 cm^2^/mL, cell donor variability, and oxygen availability showed that oxygen availability was the primary factor influencing ALP production by SCAP (64.3%, *p* < 0.0001) (Figure 2).

**FIGURE 3:**
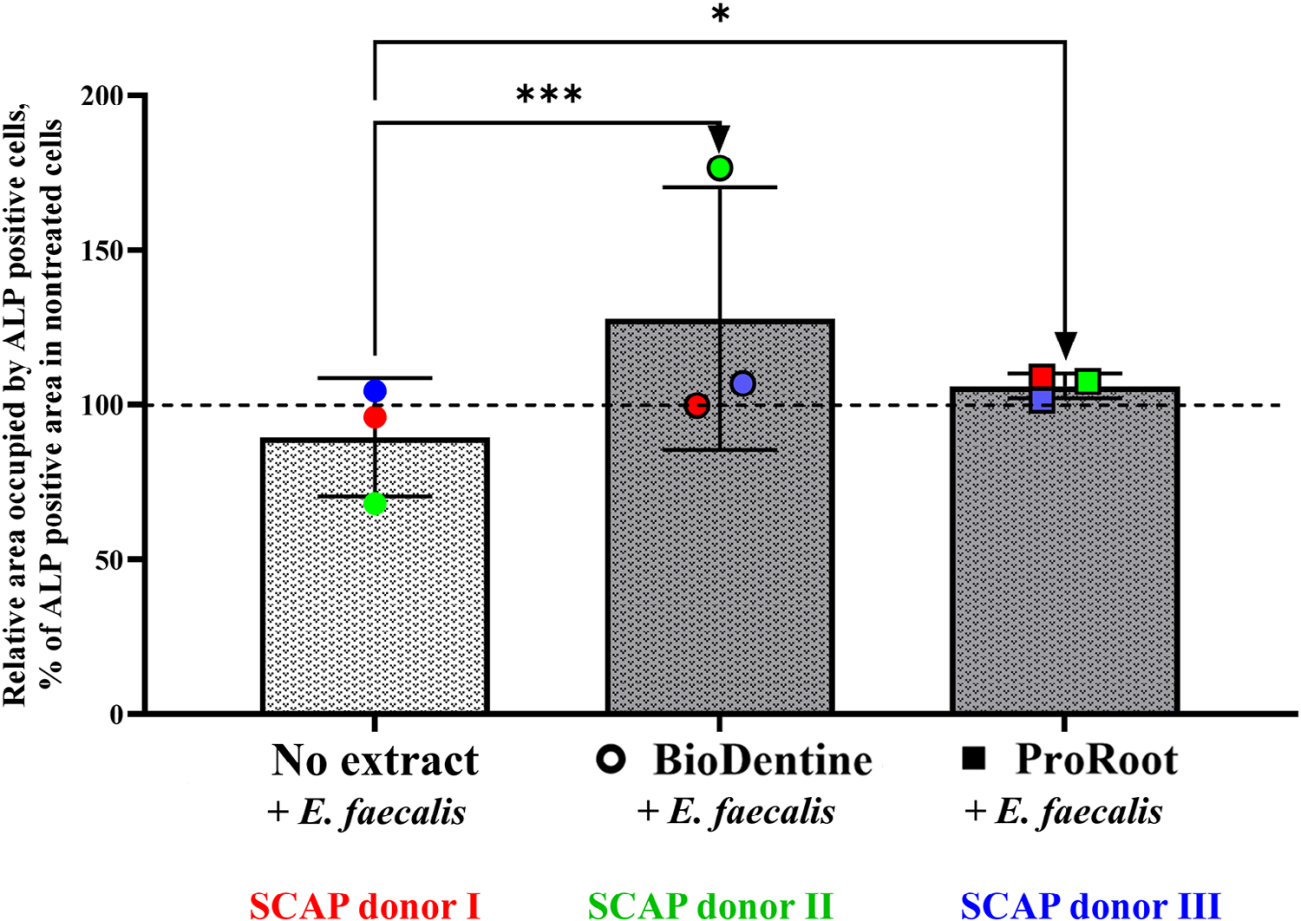
Relative area occupied by alkaline phosphatase (ALP)‐positive stem cells from the apical papilla (SCAP) cocultured with inactivated *Enterococcus faecalis* in the presence or absence of cement (ProRoot MTA and Biodentine) extracts (6 cm^2^/mL) under anaerobic conditions. Data analysis was carried out using two‐way ANOVA(31% of variability according to treatment, *p* = 0.0005)—and Dunnett's multiple comparison test.

Subsequently, culturing SCAP with cement extracts in the presence of inactivated bacteria revealed that the cell viability was significantly decreased only when SCAP were exposed to a 6 cm^2^/mL SA:V ratio of Biodentine or ProRoot extracts, under both aerobic and anaerobic conditions (Figure S3A, B). These experimental parameters were selected to explore the impact of *E. faecalis* on the osteogenic/odontogenic potential of SCAP. The results indicated a marked contrast between the effects of material exposure alone and combined exposure to inactivated *E. faecalis* and the extract. Specifically, the presence of inactivated *E. faecalis* led to a significant increase in ALP activity in SCAP cultured with Biodentine and ProRoot MTA extracts (Figure 3).

## DISCUSSION

Considering the widespread clinical application of Biodentine and ProRoot MTA, further research is essential to elucidate their effects on SCAP under varying conditions, which are fundamental and inevitable. The main goal of this study was to examine the effects of ProRoot MTA and Biodentine on the viability and mineralization of SCAP in the presence of UVC‐inactivated *E. faecalis* under both aerobic and anaerobic conditions. Our findings showed no significant difference between Biodentine and ProRoot MTA in the viability of SCAP, although higher SA:V ratios of Biodentine and ProRoot MTA were associated with increased cytotoxicity. The process of mineralization, as measured by ALP activity, appeared to be influenced more by donor‐specific factors and environmental conditions, particularly anaerobic settings, than by the type of material. Additionally, the combination of *E. faecalis* and the cement material extracts notably affected ALP production. These results suggest that although both Biodentine and ProRoot MTA have comparable effects on SCAP, the presence of *E. faecalis* and low oxygen conditions are key factors influencing cellular responses, emphasizing the need for further exploration of these variables in clinical practice.

In this study, we adhered to ISO 10,993‐12 to ensure standardized biological evaluation and enhanced reproducibility and comparability across experiments. An SA:V ratio of 3 cm^2^/mL was used to optimize the exposure conditions, and the samples were fully immersed in the culture medium during the release phase to ensure accurate assessment. This standardization minimizes variability, improves data reliability, and ensures compliance with the regulatory requirements [35]. A similar approach was utilized previously to test the influence of bioceramic materials on SCAP [40]. In accordance with our results, a high MTA concentration of 20 mg/mL was cytotoxic to cementoblasts, whereas lower concentrations did not cause changes in cell viability [41]. Additionally, it has been shown that the effect of MTA on cells is dose‐dependent, with higher concentrations inhibiting SCAP proliferation, while lower concentrations do not significantly affect the viability of SCAP. Moreover, appropriate concentrations of MTA can enhance the osteogenic/odontogenic differentiation of SCAP by activating the p38 and extracellular signal‐regulated kinase (ERK) pathways [42]. Although the two reports reached different conclusions regarding the effects of low MTA concentrations, they both agreed that higher MTA concentrations were more cytotoxic.

Regarding Biodentine, a low concentration (2 mg/mL) has been shown to stimulate the proliferation of stem cells from human exfoliated deciduous teeth and dental pulp stem cells (DPSCs), whereas a concentration of 20 mg/mL exhibits a cytotoxic effect [43, 44]. These findings are consistent with our results, suggesting that concentration, rather than cement type, determines cell cytotoxicity.

An important aspect of regenerative endodontic treatment is the potential of SCAP for odontogenic and osteogenic differentiation, promoting mineralized tissue formation and increasing dentin wall thickness [18]. Therefore, we assessed the levels of the early osteogenic/odontogenic marker (ALP) in SCAP after 21 days of incubation with solutions containing extracts from Biodentine and ProRoot MTA. It was previously indicated that calcium silicate‐based cements can induce osteogenic/odontogenic differentiation of SCAP [45]. Biodentine can also increase the levels of ALP in DPSCs after 2 weeks of coculture [46]. However, another study found that the ALP levels in DPSCs did not change significantly after 10 days of coculture with MTA or Biodentine [47]. Our results indicate that ALP levels vary because of donor‐dependent differences. As SCAP are mesenchymal stem cells (MSC), donor‐specific variability significantly affects their differentiation potential and proliferation [48].

Notably, we not only analyzed responses of SCAP to extracts from Biodentine and ProRoot MTA but also evaluated SCAP mineralization in response to both bacterial stimuli and cement extracts. We have previously demonstrated that *E. faecalis* stimulates the secretion of ALP by SCAP [49]. Consistently, this study showed that combining inactivated *E. faecalis* with a cement extract promoted SCAP mineralization. By contrast, it has been shown that activation of tumor necrosis factor alpha (TNF‐𝛼) in macrophages by common endodontic pathogens, including *E. faecalis*, decreases SCAP mineralization [50]. *Enterococcus faecalis* is strongly associated with root canal treatment failure [51, 52, 53] and is commonly associated with secondary apical diseases [54]. This highlights the importance of persistent microbiota in SCAP modulation, which can alter the effects of restorative biomaterials on SCAP and potentially impact the prognosis of regenerative treatments.

Another aspect considered in our study was the oxygen conditions, as SCAP derived from MSCs thrive in low oxygen environments, where hypoxia can modulate their differentiation [55]. Hypoxia promotes osteogenesis in MSCs by elevating the levels of osteocalcin and osteopontin compared with those present in normoxic conditions [56]. Our findings indicate that oxygen levels affect ALP production more significantly than the material type, highlighting the importance of studying root‐filling materials under anaerobic conditions to mimic the in vivo environment of the root canal.

In this study, we assessed whether the two bioactive materials had different effects on SCAP viability and mineralization, which in turn reflected their osteogenic potential. However, our results showed no significant difference between the effects of Biodentine and ProRoot MTA on SCAP. Importantly, Biodentine and ProRoot MTA have been shown to be beneficial in regenerative endodontic treatment owing to their ability to enhance SCAP mineralization [57]. A prior study has shown that ProRoot MTA and Biodentine exhibit similar properties; however, Biodentine may be more favorable because it is easier to handle and has lower discoloration potential [13].

The potential limitations of this study may have influenced its results. Specifically, the use of Biodentine extracts at the ISO‐recommended SA:V ratio of 3 cm^2^/mL for the ALP detection experiment, compared with a higher SA:V ratio of 6 cm^2^/mL, may partly explain the differences observed between the materials. Variations in cement extract SA:V ratios could influence cellular responses and should be considered when interpreting the findings. Although this study aimed to replicate conditions of immature and infected root canals during endodontic treatment, the results should be interpreted with caution because of the use of an in vitro model with a monolayer SCAP culture; this lacks the complexity of the in vivo environment, including inflammatory processes and pathogen interactions. Furthermore, because donor variability significantly influences SCAP responses, future studies should include a greater number of donors to enhance the generalizability of findings. These results highlight the importance of considering various factors, including oxygen availability, variability based on cell donors, and microbial factors, when studying the influence of materials on dental stem cells.

This study demonstrated that both Biodentine and ProRoot MTA exhibited similar effects on the viability and mineralization of SCAP in vitro. The presence of *E. faecalis* and culture under anaerobic conditions significantly influenced SCAP behavior, particularly in terms of cytotoxicity and mineralization. Although no significant difference was observed between the two materials, higher SA:V ratios of the Biodentine and ProRoot MTA extracts were associated with increased cytotoxicity, and mineralization was more dependent on environmental conditions and donor variability than on the material. These findings elucidate the importance of considering the presence of microbes and oxygen levels when evaluating the effectiveness of endodontic materials. Further in vivo studies are required to assess the clinical relevance of these results and to explore the long‐term effects of these materials under more complex conditions. Future research should investigate the molecular mechanisms underlying SCAP responses to biomaterials and microbial interactions and optimize formulations to enhance biocompatibility. By addressing these challenges, this study establishes a foundation for improving regenerative endodontic therapies and optimizing clinical outcomes.

## AUTHOR CONTRIBUTIONS

**Conceptualization**: Nelly Romani Vestman. **Formal analysis**: Olena Rakhimova. **Investigation**: Olena Rakhimova. **Methodology**: Lahood Abdalla, Bagir Soltani, and Olena Rakhimova. **Formal analysis**: Olena Rakhimova. **Writing—original draft**: Lahood Abdalla, Bagir Soltani, Olena Rakhimova, and Valeriia Zymovets. **Writing—review & editing**: Valeriia Zymovets, Peyman Kelk, Malin Brundin, and Nelly Romani Vestman.

## CONFLICT OF INTEREST STATEMENT

The authors declare no conflicts of interest related to this study.

## Supporting information



Supporting Information
