# Supplementary material for: Modulation of primary human apical papilla stem cells: Influence of Enterococcus faecalis, oxygen levels, and calcium silicate‐based cements
Source: Eur J Oral Sci. 2025 Jun 13;133(5):e70025. doi: 10.1111/eos.70025 (PMC12445809; doi:10.1111/eos.70025)
Supplement: Supplementary file 1 — Supporting Information [file EOS-133-e70025-s001.pdf]

SUPPORTING INFORMATION

Modulation of primary human apical papilla stem cells: Influence of *Enterococcus faecalis*, oxygen levels, and calcium silicate-based cements

OLENA RAKHIMOVA, VALERIIA ZYMOVETS, LAHOOD ABDALLA, BAGIR SOLTANI, MALIN BRUNDIN, PEYMAN KELK, NELLY ROMANI VESTMAN

Department of Odontology, Umeå University, Sweden.

Department of Medical and Translational Biology, Umeå University, Umeå, Sweden

Wallenberg Centre for Molecular Medicine, Umeå University, Umeå, Sweden;

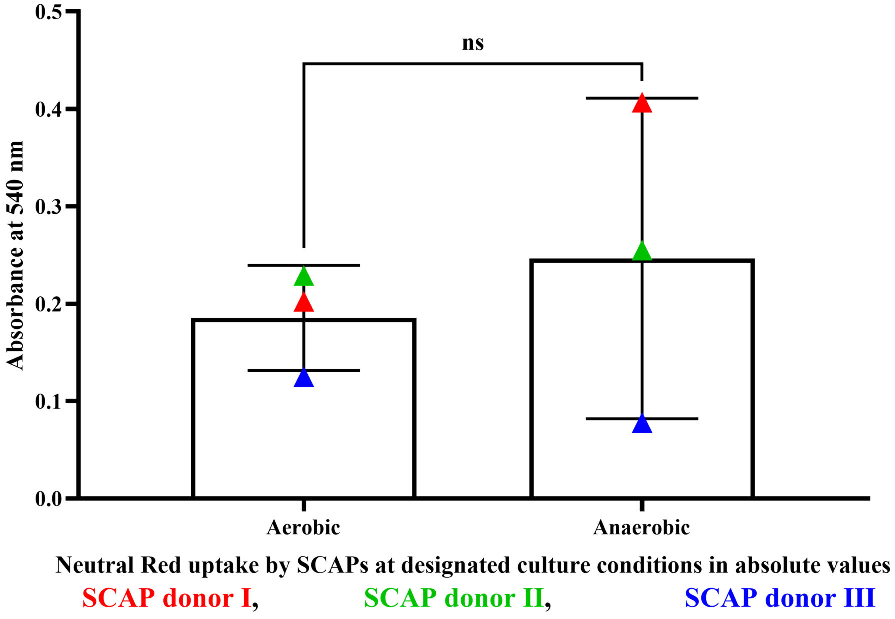

Figure S1. Neutral Red uptake in aerobic versus anaerobic conditions by SCAPs. Data analyzed by unpaired t-test (Mann Whitney version for comparison of data sets with the non-Gaussian distribution). Each bar represents mean value based on the average value of three biological replicas (SCAP donor I, -II, -III) taken in six technical replicas at each variant of treatment: Aerobic versus Anaerobic with Standard Deviation (SD). The absolute values of viability in each variant of culture were normalized to corresponding non-treated controls and expressed in percentage.

A

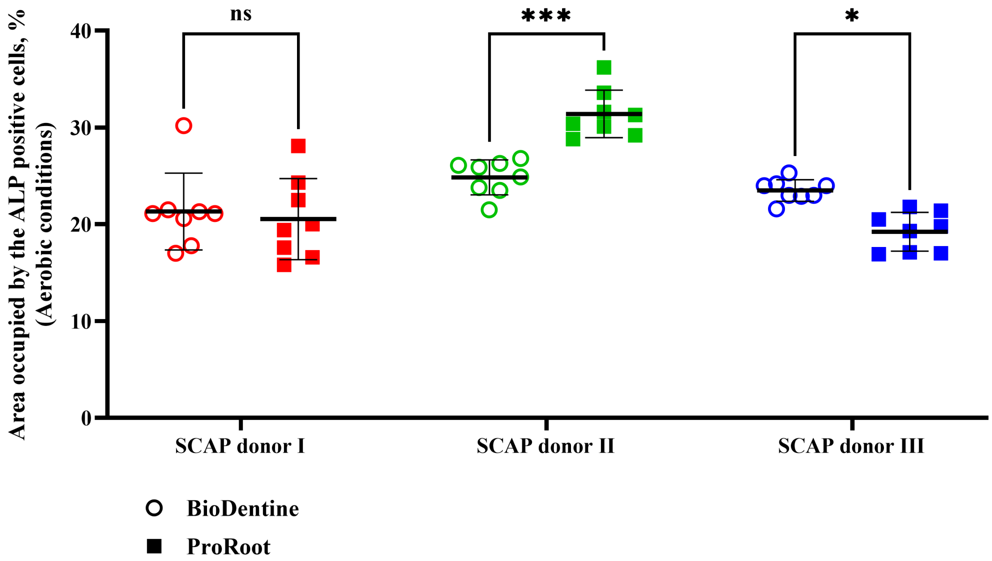

B

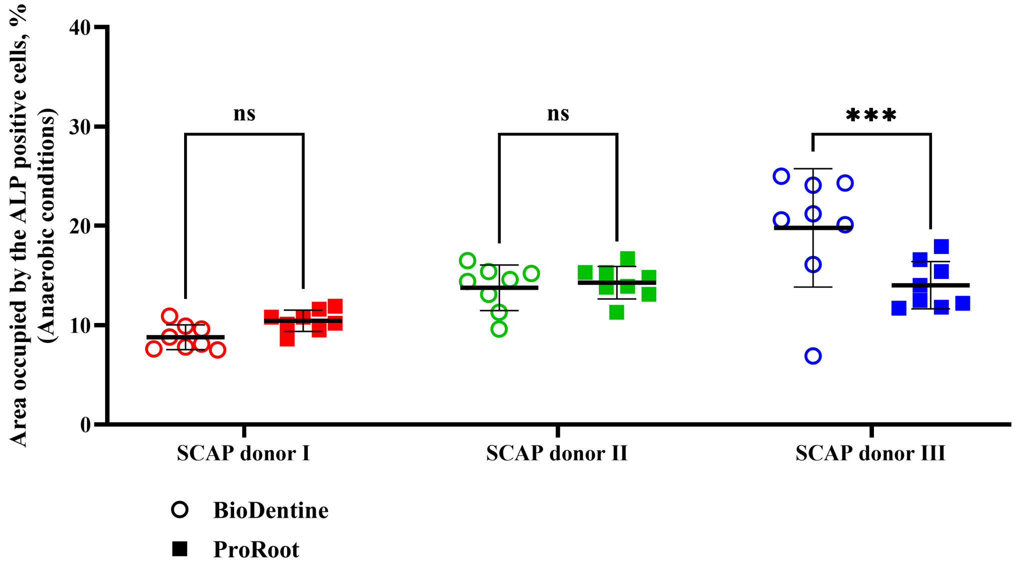

Figure S2. Area occupied by ALP-positive SCAP cells under (A) aerobic and (B) anaerobic conditions. Data were analyzed using two-way ANOVA, revealing that SCAP donor variability accounted for 47% of the total variance in aerobic conditions ($p < 0.0001$) and 46% in anaerobic conditions ($p < 0.0001$). Interaction with the type of cement contributed 22% of the variability in aerobic conditions ($p < 0.0001$) and 14% in anaerobic conditions ($p < 0.0017$). Sidak's multiple comparisons test was used to assess differences in ALP-positive cell areas between BioDentine and ProRoot cement extracts for each donor.

A

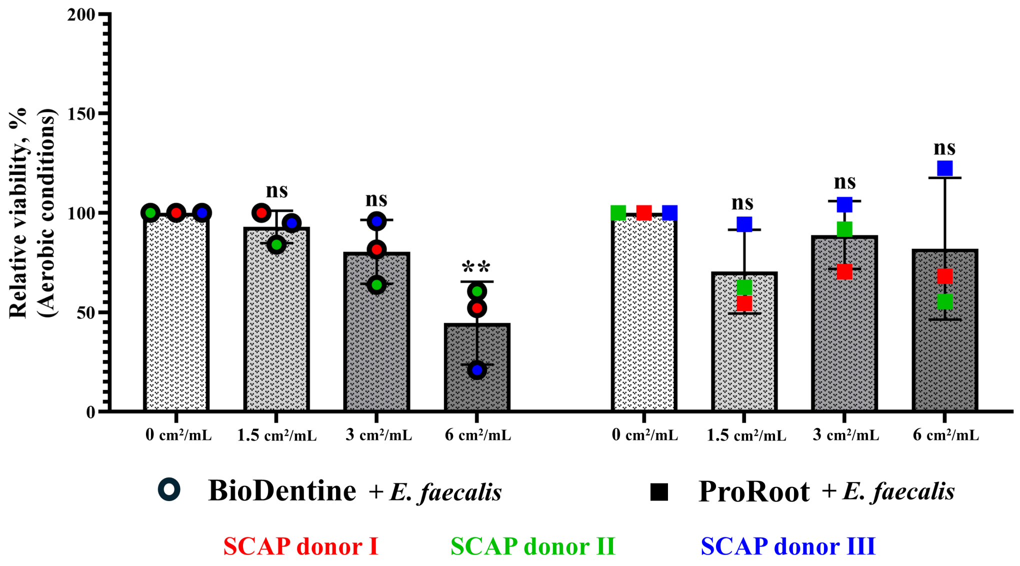

B

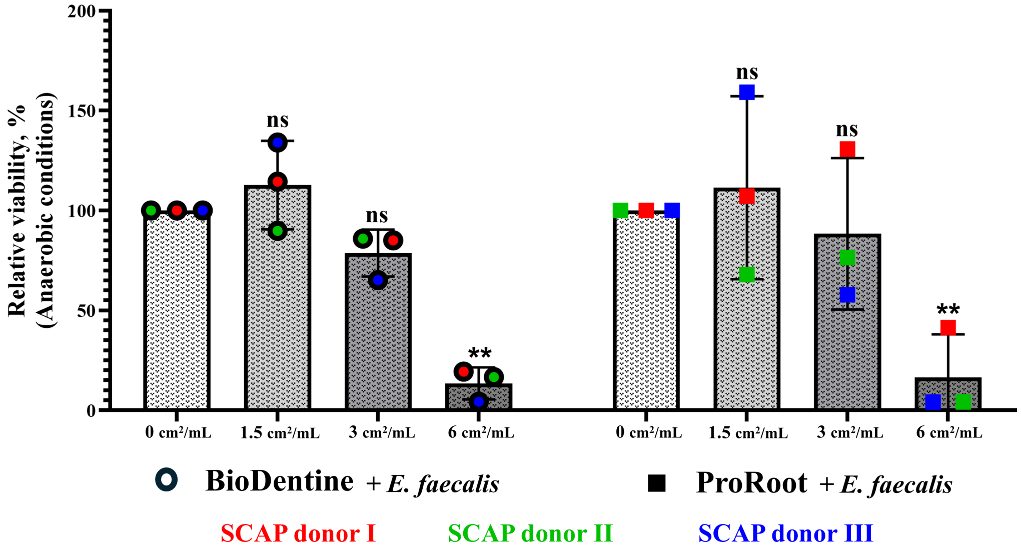

Figure S3. Relative viability of SCAP co-cultured with inactivated *E. faecalis* in the presence or absence of different concentrations of cement extracts (0 cm²/mL, 1.5 cm²/mL, 3 cm²/mL, and 6 cm²/mL) (A) aerobically or (B) anaerobically. Analysed using two-way ANOVA (variation due to cement concentration—78% ($p<0.0001$)) and followed by Sidak's multiple comparison test.

Table S1.

Šídák's multiple comparisons test of Mean values of viability of the SCAPs cultured aerobically in presence of designated concentrations of BioDentine versus MTA

Variants of comparison of SCAPs viability at designated variant of culture	Mean diff.	95,00% CI	Below threshold?	Summary	Adjusted P Value
Aerobic culture					
x0.: BioDentine vs. x0.: ProRoot MTA	0,000	-44,11 to 44,11	No	ns	>0,9999
x1/2.: BioDentine vs. x1/2.: ProRoot MTA	-19,41	-63,52 to 24,69	No	ns	0,9724
x1.: BioDentine vs. x1.: ProRoot MTA	-10,42	-54,53 to 33,69	No	ns	>0,9999
x2.: BioDentine vs. x2.: ProRoot MTA	-14,64	-58,74 to 29,47	No	ns	0,9994

Table S2.

Šídák's multiple comparisons test of Mean values of viability of the SCAPs cultured anaerobically in presence of designated concentrations of BioDentine versus ProRoot MTA.

Variants of comparison of SCAPs viability at designated variant of culture	Mean diff.	95,00% CI	Below threshold?	Summary	Adjusted P Value
Anaerobic culture					
x0.: BioDentine vs. x0.: ProRoot MTA	0,000	-51,43 to 51,43	No	ns	>0,9999
x1/2.: BioDentine vs. x1/2.: ProRoot MTA	-9,590	-61,02 to 41,84	No	ns	0,9974
x1.: BioDentine vs. x1.: ProRoot MTA	-38,78	-90,22 to 12,65	No	ns	0,2212
x2.: BioDentine vs. x2.: ProRoot MTA	-10,69	-62,12 to 40,74	No	ns	0,9950
